# Clinical implications of AR alterations in advanced prostate cancer: a multi-institutional collaboration

**DOI:** 10.1038/s41391-024-00805-3

**Published:** 2024-02-22

**Authors:** Zeynep B. Zengin, Nicholas C. Henderson, Joseph J. Park, Alicia Ali, Charles Nguyen, Clara Hwang, Pedro C. Barata, Mehmet A. Bilen, Laura Graham, George Mo, Deepak Kilari, Abhishek Tripathi, Matthew Labriola, Shoshana Rothstein, Rohan Garje, Vadim S. Koshkin, Vaibhav G. Patel, Michael T. Schweizer, Andrew J. Armstrong, Rana R. McKay, Ajjai Alva, Tanya Dorff

**Affiliations:** 1 Department of Medical Oncology & Therapeutics Research, City of Hope Comprehensive Cancer Center, Duarte, CA USA; 2 Department of Biostatistics, University of Michigan, Ann Arbor, MI USA; 3 Division of Hematology and Oncology, Department of Medicine, University of Michigan, Ann Arbor, MI USA; 4 Division of Hematology/Oncology, Department of Internal Medicine, Henry Ford Health System, Detroit, MI USA; 5 Tulane Cancer Center, Tulane University, New Orleans, LA USA; 6 Winship Cancer Institute of Emory University, Atlanta, GA USA; 7 University of Colorado Anschutz Medical Campus, Aurora, CO USA; 8 University of Washington/Fred Hutchinson Cancer Center, Seattle, WA USA; 9 Department of Medicine, Froedtert Cancer Center, Medical College of Wisconsin, Milwaukee, WI USA; 10 Stephenson Cancer Center, Oklahoma City, OK USA; 11 Division of Medical Oncology, Duke University Medical Center, Duke Cancer Institute Center for Prostate and Urologic Cancers, Duke University, Durham, NC USA; 12 Karmanos Cancer Institute, Wayne State University, Detroit, MI USA; 13 Holden Comprehensive Cancer Center, Iowa City, IA USA; 14 Division of Hematology and Oncology, Department of Medicine, University of California San Francisco, San Francisco, CA USA; 15 Icahn School of Medicine at Mount Sinai, New York, NY USA; 16 Arvinas Inc, New Haven, CT USA; 17 Moores Cancer Center, University of California San Diego, La Jolla, CA USA

**Keywords:** Predictive markers, Cancer genetics, Prostate cancer, Outcomes research

## Abstract

**Background:**

*AR* gene alterations can develop in response to pressure of testosterone suppression and androgen receptor targeting agents (ARTA). Despite this, the relevance of these gene alterations in the context of ARTA treatment and clinical outcomes remains unclear.

**Methods:**

Patients with castration-resistant prostate cancer (CRPC) who had undergone genomic testing and received ARTA treatment were identified in the Prostate Cancer Precision Medicine Multi-Institutional Collaborative Effort (PROMISE) database. Patients were stratified according to the timing of genomic testing relative to the first ARTA treatment (pre-/post-ARTA). Clinical outcomes such as time to progression, PSA response, and overall survival were compared based on alteration types.

**Results:**

In total, 540 CRPC patients who received ARTA and had tissue-based (*n* = 321) and/or blood-based (*n* = 244) genomic sequencing were identified. Median age was 62 years (range 39−90) at the time of the diagnosis. Majority were White (72.2%) and had metastatic disease (92.6%) at the time of the first ARTA treatment. Pre-ARTA genomic testing was available in 24.8% of the patients, and *AR* mutations and amplifications were observed in 8.2% and 13.1% of the patients, respectively. Further, time to progression was longer in patients with *AR* amplifications (25.7 months) compared to those without an *AR* alteration (9.6 months; *p* = 0.03). In the post-ARTA group (*n* = 406), *AR* mutations and *AR* amplifications were observed in 18.5% and 35.7% of the patients, respectively. The most common mutation in post-ARTA group was *L702H* (9.9%).

**Conclusion:**

In this real-world clinicogenomics database-driven study we explored the development of *AR* alterations and their association with ARTA treatment outcomes. Our study showed that *AR* amplifications are associated with longer time to progression on first ARTA treatment. Further prospective studies are needed to optimize therapeutic strategies for patients with *AR* alterations.

## Introduction

Prostate cancer is the most commonly diagnosed non-cutaneous cancer among men in the United States, with an estimated incidence of 268,000 cases annually [1]. Suppression of the androgen signaling pathway has been the mainstay of treatment of prostate cancer since the landmark discovery by Huggins and Hodges of the androgen dependence of prostate cancer in 1941 [2]. In the setting of advanced or metastatic disease, treatment is focused on reducing androgen levels with surgical orchiectomy or medical castration and blocking androgen receptor activation [3, 4]. While multiple treatment agents have been developed, outcomes differ widely, and validated biomarkers for treatment response or resistance are needed to help guide treatment selection.

The growing ease and affordability of genomic profiling have enabled more prostate cancer patients to undergo blood-based and/or tissue-based genomic sequencing. Approval of agents such as olaparib, the first biomarker selected therapy for prostate cancer, has catapulted us into the modern era of broad-scale sequencing for all men with advanced disease [5]. These approaches have, in turn, provided a foundation for a body of literature seeking to identify actionable alterations and predictive genomic signatures [6, 7]. The most commonly altered genes in primary prostate cancer are ETS fusions, *PTEN*, *SPOP*, *TP53*, and *FOXA1* [8]. Furthermore, in the metastatic castration resistant disease setting, alterations in *AR, TP53*, *MYC, ZMYM3*, *APC*, and *RB1* are reported to be significantly enriched compared to primary prostate cancer [9]. Although the predictive role of many of these genomic alterations is still being explored, there is heterogeneity in interpretating the clinical significance of *AR* gene alterations in patients with advanced disease.

The *AR* gene encodes a nuclear receptor that is activated following the binding of androgenic hormones and regulates the transcription of several growth and differentiation-related genes required for the survival of prostatic cells [10]. Indeed, the majority of prostate cancers are driven by the alterations in *AR*-signaling [4, 11]. Affected *AR* gene domain types, including mutations in the ligand binding domain, gene body and/or enhancer amplifications, or intronic structural rearrangements resulting in alternative splicing, can yield distinct biological characteristics and could potentially underlie the differential treatment response observed with AR targeted agents (ARTA).

*AR* gene amplifications primarily occur in response to androgen deprivation therapy and are observed in more than 50% of patients with castration-resistant prostate cancer [12–14]. *AR* amplifications have been associated with resistance to enzalutamide and abiraterone acetate treatment [15–18]. Among non-metastatic prostate cancer patients, *AR* gene anomalies, while less common, have been associated with poorer clinical outcomes [19].

Several *AR* mutations have been implicated in therapeutic resistance to ARTA, often through *AR* activation by glucocorticoids and other sex hormones (e.g. *L702H*, *H875Y*) or gain of function mutations that confer AR agonist activity to AR antagonists (e.g., *T878A*, *H875*, *F877L*, *W742C*) [12, 17], Discrepancies in the current literature, as well as the conflicting results from various genomic analyses, further complicates effective, evidence-based treatment selection among patients with prostate cancer. Given the existing knowledge gaps in the clinical relevance of AR alterations in relation to ARTA use, we embarked on our current study. In this study by utilizing a multi-institutional clinical and genomic database, we examined the association between clinical outcomes and *AR* alteration types, as well as the time of occurrence of alterations relative to the systemic treatment.

## Methods

### Study design and patient selection

A retrospective analysis was conducted using the Prostate Cancer Precision Medicine Multi-Institutional Collaborative Effort (PROMISE) database, which includes deidentified clinical and genomic data from patients with advanced prostate cancer (metastatic hormone sensitive or castration resistant) treated at 18 academic centers [20]. Patients had germline and somatic genomic testing (tissue, blood and/or germline) through CLIA certified commercially available platforms during routine clinical care. Detailed information of the assays can be found in supplementary table 1. Data was collected from registered institutions between 4/1/2020 and 7/7/2021 using a standardized RedCap database. Only genomic alterations that were reported by the genomic testing platform was included for the analysis of this study. Raw genomic data was unavailable. This study was approved by local institutional review boards at participating sites per institutional policy and the Declaration of Helsinki.

**Fig. 1:**
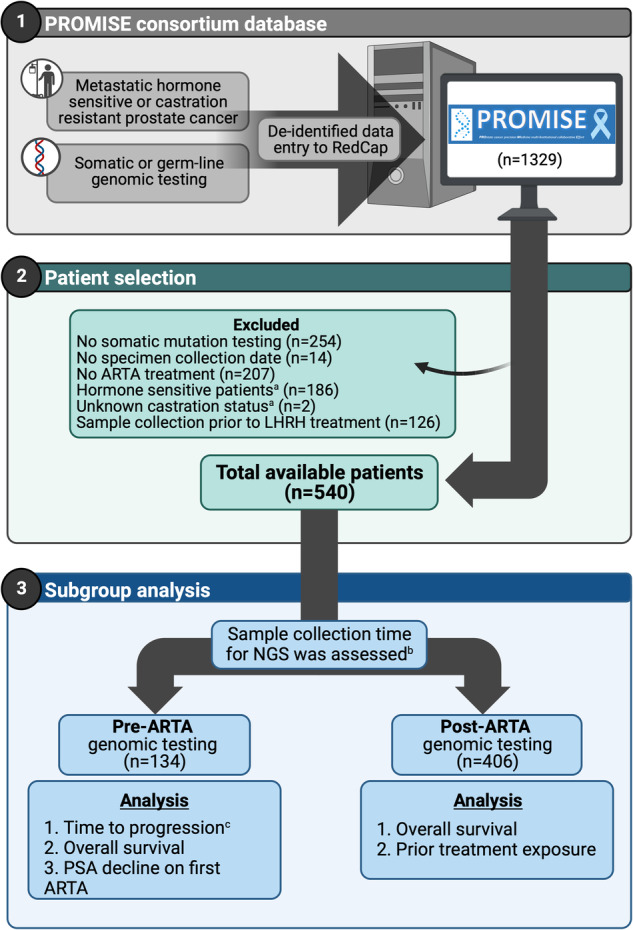
Patient selection. ^a^Castration status was determined at the first ARTA treatment initiation. ^b^For patients who had both germline and somatic testing, only somatic testing was used. ^c^Time to progression, which was defined as initiation of first ARTA to treatment discontinuation. Abbreviations: AR androgen receptor, ARTA AR targeted agent, PSA prostate specific antigen, NGS next generation sequencing. Image was created with Biorender.com.

For the purpose of this study, we included castration resistant prostate cancer (CRPC) patients at the time of the first ARTA treatment and had available somatic genomic testing performed on blood or tissue (Fig. 1). Only samples that were collected after androgen deprivation therapy exposure were included in this study. Subjects not meeting these criteria or for whom these details could not be verified were excluded. Patients were then categorized as pre-ARTA or post-ARTA depending on whether the genomic sequencing sample was acquired before or after exposure to ARTA. Eligible patients received any ARTA, including abiraterone, apalutamide, darolutamide or enzalutamide. Gonadotropin-releasing hormone agonist/antagonist therapy and first-generation antiandrogens were not classified as ARTAs in this study.

Cases were individually reviewed by a genomics expert in a multi-step quality control process. The RedCap database was queried for all patients marked as having an AR alteration. In a second step, to ensure all cases had been identified, the free text field containing the raw next generation sequencing (NGS) report (excluding protected health information) was separately queried to identify any unmarked AR alterations. At each institution at least 10% of the entries were quality controlled by a physician with a genomic expertize. Patients who had insufficient tumor for analysis or incomplete/missing NGS reports were excluded. Entries needing clarification were flagged and returned to the site for query resolution. After this two-stage process, a second genomics expert at the central site was consulted regarding any unclear entries.

### Outcome measures

Data regarding patient demographic characteristics, PSA values, treatment types, genomic profile and assay type were extracted from the database. In patients with more than one genomic testing, only the first somatic genomic testing was included for the analysis.

For patients in the pre-ARTA NGS group, the outcome measures of interest were PSA decline of ≥50%, time to progression (TTP), and overall survival (OS). Further, these outcome measures were compared based on *AR* alteration status (no alteration vs mutation or amplification). In the post-ARTA NGS group, the frequency of specific AR alterations (*L702H*, *T878A*, *H875Y*, *W742C*) was evaluated for their association with prior treatment exposures by the time of genomic testing and OS. TTP was defined as the time from the initiation of ARTA to treatment discontinuation due to clinical progression or censored at the time of the last documented follow-up. OS was calculated from the time of initiation of ARTA to death or censored at the time of the last follow-up.

### Statistical methods

Descriptive statistics of patient characteristics and outcomes were described using proportions for categorical variables and median and interquartile range for continuous variables. Median TTP during the first ARTA treatment and median OS were estimated using the Kaplan-Meier method. The log-rank test was used to assess the differences in TTP and OS across subgroups, as defined by *AR* alteration status.

## Results

### Patient characteristics

In total, 540 patients with CRPC who received an ARTA and had tissue (*n* = 321) and/or blood (*n* = 244) sample collection for genomic testing following androgen deprivation therapy exposure were identified using the PROMISE database. Median age was 62 (range: 39−90) years, and 55.2% had a Gleason score of ≥8 at the time of the diagnosis (Table 1). Among the entire population, the majority of patients were categorized as metastatic (92.5%; *n* = 500), and 7.4% (*n* = 40) had non-metastatic CRPC at the time of the first ARTA treatment.

**Table 1:** Patient characteristics.

	Overall (*n* = 540)	
	Pre-ARTA (*n* = 134)	Post-ARTA (*n* = 406)
Median age at diagnosis, years (range)	62 (39−90)	61 (43−89)
Disease status–no.(%)		
Non-metastatic CRPC	7 (5.2)	33 (8.1)
Metastatic CRPC	127 (94.8)	373 (91.9)
Race–no.(%)		
White	98 (73.1)	292 (71.9)
African America	25 (18.7)	81 (20.0)
Other	11 (8.2)	33 (8.1)
Ethnicity–no.(%)		
Hispanic or Latin	4 (3.0)	17 (4.2)
Non-Hispanic or Latino	130 (97.0)	389 (95.8)
Gleason Score–no.(%)		
6	7 (5.2)	17(4.2)
7	22 (16.4)	97 (23.9)
8 to 10	80 (59.7)	218 (53.7)
Unknown	25 (18.7)	74 (18.2)
Genomic sequencing–no.(%)		
Primary Tissue	42 (31.3)	30 (7.4)
Metastatic Tissue	60 (44.8)	189 (46.6)
Blood	41 (30.6)	203 (50.0)
AR alteration status–no.(%)		
AR mutations	11 (8.2)	75 (18.5)
AR amplifications	27 (13.1)	145 (35.7)
No AR alteration	96 (71.6)	200 (49.3)

*AR* Androgen receptor, *ARTA* AR targeting agent, *CRPC* Castration-resistant prostate cancer.

Pre-ARTA genomic testing was available in 24.8% (134/540) of patients, while post-ARTA genomic sequencing was available in 75.2% (406/540) of the patients. *AR* amplifications and *AR* mutations were observed in 31.8% (*n* = 172) and 15.9% (*n* = 86) of the patients, respectively. Of these, the majority of amplifications (84.3%; *n* = 145) and mutations (87.2%; *n* = 75) were from post-ARTA samples. Detailed characteristics of the study cohort are summarized in Table 1.

### Genomic testing prior to ARTA (pre-ARTA)

Pre-ARTA genomic testing was available from 134 (24.8%) patients treated with ARTA for CRPC. Of these, 94.8% (*n* = 127) of patients had metastatic CRPC, whereas 5.2% (*n* = 7) had non-metastatic CRPC at the time of the first ARTA treatment. *AR* mutations and amplifications were identified in 11 (8.2%) and 27 (13.1%) patients, respectively (Table 1). The most commonly observed AR mutation in the pre-ARTA group was L702H (*n* = 4) followed by H875Y and W743C (*n* = 3 for each). Notably, in patients with AR alterations pre-ARTA there were none who had both mutation and amplification. Most common co-occurring alterations in the *AR* mutated group were seen in *PTEN, MSH2*, and *PIK3CA* genes (18.2% each) and in patients with *AR* amplification *TP53* (48.1%), *PTEN* (25.9%), *TMPRSS2* (22.2%) and *MCL1* (22.2%) alterations were common. Overall, the top 3 most commonly occurring genomic alterations were the same between AR altered and AR non-altered patients; TP53, PTEN and TMPRSS2 with similar frequencies (33.3%, 18.8%, and 15.6%respectively).

PSA decline of ≥50% compared to baseline following ARTA treatment were observed in 90.0% of the *AR* mutated patients, 70.6% of the patients with *AR* amplification (Table 2). Compared to the patients with no *AR* alteration (76.3%), there was no statistical difference noted in the PSA response for *AR* mutated and amplified patients (*p* = 0.57 and *p* = 0.85, respectively). Median OS was also not significantly different among CRPC patients with or without *AR* alterations.

**Table 2:** Characteristics and analysis of response to ARTA in CRPC patients with pre-ARTA genomic testing.

		No AR alterations (No AR) *n* = 96	AR mutations (ARm) *n* = 11	AR amplifications (ARa) *n* = 27	*P* value (multiplicity-adjusted *p* value)	
					Arm vs. No AR	Ara vs. No AR
PSA decline ≥ 50% *n* (%)		58 (76.3)	9 (90.0)	12 (70.6)	0.57 (1.0)	0.85 (1.0)
Median TTP months (95% CI)	Overall (*n* = 134)	9.6 (6.6−21.8)	9.6(7.7 – NR)	25.7 (8.8 – NR)	0.61 (1.0)	0.03 (0.34)
	Abiraterone (*n* = 77)	11.0 (7.2−21.8)	9.6 (7.7 – NR)	18.3 (8.8-NR)	0.36 (1.0)	0.18 (1.0)
	Enzalutamide/Apalutamide (*n* = 55)	9.3(5.7−24.5)	6.8 (6.8-NR)	41.8 (2.8 – NR)	0.53 (1.0)	0.07 (0.74)
Median overall survival months (95% CI)		32.9 (26.3−42.6)	38.2 (38.2-NR)	30.8 (20.9-NR)	0.28 (1.0)	0.87 (1.0)

*AR* Androgen receptor, *TTP* time to progression.

Compared to patients without an *AR* alteration (9.6 months [95% CI 6.6−21.8]), median TTP on first ARTA in patients with *AR* amplifications (25.7 months [8.8-NR]) was significantly longer (*p* = 0.03; Table 2). No significant difference between patients with *AR* mutation (9.6 months [95% CI 7.7-NR]) and without an *AR* alteration was noted (*p* = 0.36).

### Genomic testing after ARTA (Post ARTA)

Post-ARTA genomic testing was available from 406 (75.2%) CRPC patients, with 91.9% (*n* = 373) of this group possessing metastatic disease. Median lines of prior treatment exposure in post-ARTA group were 3 (range 1−10). *AR* mutations and amplifications were seen in 18.5% (*n* = 75) and 35.7% (*n* = 145) of the patients, respectively and 14 patients (3.4%) had both types of alterations. The most common *AR* mutations in the post-ARTA group were *L702H* (9.9%, 40/406), followed by *T878A* (5.9%, 24/406), regardless of prior treatment type. The most commonly received treatments among patients with *T878A*, *W742C*, *L702H*, and *H875Y* were abiraterone and enzalutamide (Table 3). *W742C* and *L702H* mutations were more commonly seen after one line of ARTA, whereas the other mutations showed greater prevalence after exposure to a second ARTA. *AR* mutations in the post-ARTA group were observed at similar rates in patients with prior docetaxel exposure (0.14%) compared to those without prior exposure (19.9%; *p* = 0.29).

**Table 3:** Treatment exposure patterns in post-ARTA NGS group.

	L702H *n* = 40	T878A *n* = 24	H875Y *n* = 13	W742C *n* = 5
Prior treatment exposures– no (%)				
Abiraterone	34 (85.0)	23 (95.8)	11 (84.6)	2 (40.0)
Apalutamide	2 (5.0)	2 (8.3)	0 (0.0)	0 (0.0)
Bicalutamide	14 (35.0)	7 (29.2)	5 (38.5)	0 (0.0)
Cabazitaxel	16 (40.0)	7 (29.2)	3 (23.1)	0 (0.0)
Docetaxel	19 (47.5)	10 (41.7)	5 (38.5)	1 (20.0)
Enzalutamide	33 (82.5)	11 (45.8)	11 (84.6)	4 (80.0)
Number of prior ARTA exposure – no (%)				
1	14 (35.0)	14 (58.3)	4 (30.8)	4 (80.0)
2	24 (60.0)	6 (25.0)	8 (61.5)	1 (20.0)
3 +	2 (5.0)	4 (16.7)	1 (7.7)	0 (0.0)

**Fig. 2:**
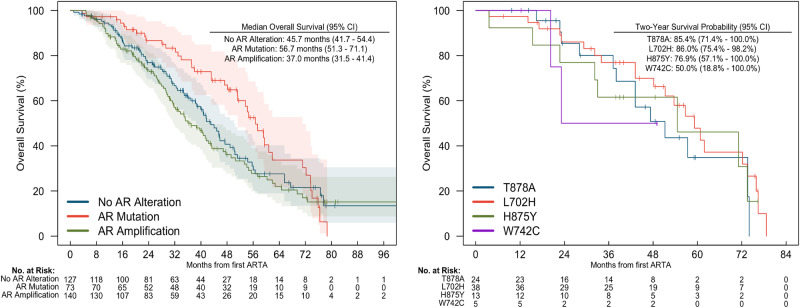
Overall Survival in post-ARTA group based on *AR* alteration types.

Overall survival for patients with *AR* alterations detected post-ARTA is shown in Fig. 2. 2-year landmark OS was 85.4% (95% 71.4−100.0) in patients with T878A mutations, 86.0% (95% 75.4−98.2) among patients with L702H mutations, 76.9% (95% 57.1−100.0) in patients with H875Y mutations, and 50% (95% 18.8−100.0) in patients with W742C mutations.

## Discussion

Alterations in *AR* gene have long been noted as drivers in the progression of prostate cancer to its castration-resistant state. The increased availability of next generation sequencing and the advent of liquid biopsy has led to several publications outlining evolution of AR alterations during the course of prostate cancer treatment [21, 22]. However, many studies have lacked granular clinical data in order to clearly define the association of AR alterations with specific treatment outcomes. Genomic reports often indicate whether benefit or lack of benefit is expected with various ARTA in the context of AR mutations or amplification, yet there are not enough data to clearly determine this, which is reflected in the conflicting recommendations seen in reports from different next generation sequencing providers. The PROMISE database, which included 1329 prostate cancer patients who had genomic testing data linked to detailed clinical treatment information, provides a unique opportunity to add new data to this area of uncertainty.

Prior studies evaluating the impact of *AR* mutations on treatment outcomes have been small and/or included patients with prior ARTA exposure, since this is the group in which these mutations are most frequently seen. In our dataset we were able to identify 11 patients with *AR* mutations and 27 patients with *AR* amplifications that arose prior to any ARTA exposure. Our findings yielded the novel insight that *AR* amplifications are associated with longer time to clinical progression on first ARTA treatment compared to patients without an *AR* alteration. Whereas, in patients with *AR* mutation median TTP was similar to those without an *AR* alteration. Similar to ours, in one study which only a single genomic testing platform was used, median TTP in pre-ARTA mCRPC patients with ligand binding domain mutations was 6.2 months [23]. However, AR amplifications in this cohort was noted to be associated with poor prognosis. Further a shorter TTP trend in the group with higher copy number alterations was seen. Although it should be noted that our sample size is small, and these results might be driven by some outliers in our dataset.

Several studies have demonstrated that patients with *AR* amplifications had worse clinical outcomes with ARTA treatment, however the majority of these studies included a mixed cohort of patients who were exposed to prior ARTA [15, 16, 24–26]. Further, in these studies, liquid biopsy was used to determine *AR* alterations. Since *AR* copy number gain is correlated with higher cell-free DNA levels which are often associated with higher tumor volume, liquid biopsy can create a confounding effect for the prognostic value of *AR* amplifications [27]. Similarly in our study, liquid biopsy was used in 40% of the patients and might be creating a similar confounding effect in this group. In another study, Jayaram and colleagues looked at a cohort from PCR2023 study (ClinicalTrials.gov identifier: NCT01867710) which included 133 baseline liquid biopsy samples of treatment naïve CRPC patients who were randomized to receive abiraterone acetate and one of four different glucocorticoid regimens [27]. Patients with *AR* copy number above ≥1.92 (*n* = 22) had shorter PFS and OS compared to those with *AR* copy number of <1.92. Although the increased copy number gain in this study was associated with worse clinical outcomes, authors did not use patients without an *AR* alteration as the comparator arm, making it hard to interpret the outcomes for these groups.

*AR* mutations remain infrequent among treatment naïve prostate cancer patients but are detected in almost 20% of those with castration resistant disease, and in up to 40% of patients who have received ARTA [12, 28]. There did not appear to be a unique genomic landscape among patients who develop AR alterations at castration resistance without prior ARTA compared to those who develop AR alterations after exposure to ARTA. It should be also noted that, in the current landscape where ARTA is being used in the metastatic hormone sensitive prostate cancer, we may see earlier emergence of these AR alterations and impact of these on other types of treatments used for mCRPC has not yet been explored. Similar to published literature, in our study cohort with castration resistant prostate cancer patients we found an increased rate of *AR* mutation frequency among those who received ARTA (19%) compared to those who did not (8%) as well as co-occurrence of AR amplification together with mutation which was not seen pre-ARTA exposure. In line with previous studies, the most common mutation in post-ARTA genomic reports in our study was *L702H*, which occurred with similar rates post abiraterone or enzalutamide [16]. In previous studies, this mutation was shown to be associated with resistance to abiraterone, and further androgen receptor signaling was shown to be activated by prednisone or progesterone in the presence of this mutation [17, 29, 30]. Although the numbers were small in our cohort, *L702H* was associated with higher 2-year OS compared to *H875Y* and *W742C*, which may imply better behavior or greater responsiveness to subsequent lines of therapy. Nevertheless, future drug development to target *L702H* will be critical since it is the most common, and the current AR degraders such as ARV-110 may be less effective in this subset [31].

Strengths of this analysis include having multiple commercial assays in a wide geographic range of prostate cancer patients being treated with standard therapies as opposed to restricting analysis to patients from one center and using one assay. This may increase the clinical applicability. Furthermore, genomic review by an expert ensured high quality data is the most critical aspect of this precision medicine effort.

The current study represents real-world practice, and as such sample collection sites, genomic testing platforms and their associated methodologies were heterogenous. The sample size was limited by not all patients having a genomic testing following ARTA treatment. As a result, temporal and spatial heterogeneity, as well as differences in assay methodologies, lack of transcriptomic, and splice variant analysis may have limited the ability to identify all relevant alterations. In our study we used multiple commercial assays which is the regular clinical practice and these tests have different coverages and sequencing technologies causing variations in the results. These tests are also usually pan tumor and not prostate cancer specific and thus may not be designed to optimally detect all AR alterations or ETS rearrangements. Lastly, in real world setting, getting sequencing once either with liquid biopsy or archived tissue is common practice. This makes the results impactful and relevant to clinical practice even though the prior studies may have reported higher AR alteration prevalence [13, 14]. Additionally, imaging practices were not uniform throughout this retrospective database, thus making the use of validated endpoints, including radiographic progression-free survival, impossible to ascertain. Despite this, we were able to examine overall survival among our study cohort along with clinically relevant endpoints such as PSA decline and time to treatment change, providing meaningful insight into disease progression. Future research efforts in this domain should include serial testing and enhanced inclusion of patients treated with specific therapies, as well as increasing the number of patients registered in databases such as PROMISE.

## Conclusions

The current study utilized the PROMISE database to gain novel insight into the impact of AR amplifications and mutations on treatment response to ARTA. These findings highlight the need for prospective data or further rigorous database analyses to truly define whether treatment decision making should be impacted by detection of AR alteration on next-generation sequencing. Such steps will advance the potential for personalized medicine, and access to next generation sequencing platforms in advanced prostate cancer will remain central to its success.

## Supplementary information

Supplementary table 1

## Data Availability

The datasets of the current study are not publicly available due to institutional review board restrictions.
