# Supplementary material for: Clinical implications of AR alterations in advanced prostate cancer: a multi-institutional collaboration
Source: Prostate Cancer Prostatic Dis. 2024 Feb 22;28(2):378–84. doi: 10.1038/s41391-024-00805-3 (PMC12106060; doi:10.1038/s41391-024-00805-3)
Supplement: Supplementary file 1 — Supplementary table 1 [file 41391_2024_805_MOESM1_ESM.docx]

**Supplementary Table 1.** Germline and somatic testing platforms

| **Site** | **NGS Assays** | **Tissue** | **Blood (cfDNA or CTC)** | **Blood or Saliva (Germline)** | **Somatic** | **Germline** | **Number of Genes** |
| --- | --- | --- | --- | --- | --- | --- | --- |
| **University of Michigan** | Foundation One CDx | X |  |  | X |  | 324 |
|  | Guardant360 CDx |  | X |  | X |  | 74 |
|  | MiOncoseq (in-house) | X |  |  | X |  | 1711 |
|  | Tempus xF |  | X |  | X |  | 105 |
| **Winship Cancer Institute  Emory University** | Ambry (unspecified) |  |  | X |  | X | N/A |
|  | Caris | X |  |  | X |  | 592 |
|  | Foundation One CDx | X |  |  | X |  | 324 |
|  | Guardant360 CDx |  | X |  | X |  | 74 |
|  | Invitae Germline (unspecified) |  |  | X |  | X | N/A |
| **Moores Cancer Center University of California San Diego** | Ambry CustomNext (genes selected by clinician) |  |  | X |  | X | Up to 91 |
|  | Foundation One CDx | X |  |  | X |  | 324 |
|  | Guardant360 CDx |  | X |  | X |  | 74 |
|  | Invitae Common Hereditary Cancer Panel |  |  | X |  | X | 47 |
|  | Invitae 83 gene panel |  |  | X |  | X | 83 |
|  | Invitae 134 gene panel |  |  | X |  | X | 134 |
|  | Invitae 156 gene panel |  |  | X |  | X | 156 |
|  | Invitae Germline (unspecified) |  |  | X |  | X | N/A |
|  | Invitae Multi-Cancer Panel |  |  | X |  | X | 84 |
|  | Invitae Prostate Cancer Panel (19 gene panel) |  |  | X |  | X | 19 |
|  | Tempus xF (cfDNA) |  | X |  | X |  | 105 |
|  | Tempus xO (tissue) | X |  | X | X | X | 1711 |
|  | Tempus xT (595 gene panel) | X |  |  | X |  | 595 |
|  | Tempus xT (648 gene panel) | X |  |  | X |  | 648 |
|  | Other | X |  |  | X |  | N/A |
| **Fred Hutchinson Cancer Center University of Washington** | BROCA Cancer Risk Panel |  |  | X |  | X | 74 |
|  | Color Germline (unspecified) |  |  | X |  | X | N/A |
|  | Color Hereditary Cancer Test |  |  | X |  | X | 30 |
|  | FoundationACT |  | X |  | X |  | 62 |
|  | Foundation One CDx | X |  |  | X |  | 324 |
|  | Myriad Germline (unspecified) |  |  | X |  | X | N/A |
|  | Oncoplex (tissue) | X |  |  | X |  | > 350 |
|  | Oncoplex (CTC) |  | X |  | X |  | > 350 |
|  | Stand Up 2 Cancer | X |  |  | X |  | N/A |
| **Duke Cancer Institute Duke University** | Ambry (unspecified) |  |  | X |  | X | N/A |
|  | Foundation One pre-CDX | X |  |  | X |  | N/A |
|  | Foundation One CDX | X |  |  | X |  | 324 |
|  | Foundation One Liquid CDx |  | X |  | X |  | 70 |
|  | GeneDx |  |  | X |  | X | N/A |
|  | Guardant360 |  | X |  | X |  | 74 |
|  | Invitae Germline (unspecified) |  |  | X |  | X | N/A |
|  | Invitae Multi-Cancer Panel |  |  | X |  | X | 84 |
|  | Invitae Common Hereditary Cancers Panel |  |  | X |  | X | 47 |
|  | Invitae Custom Cancer Panel |  |  | X |  | X | Variable |
|  | Strata NGS | X |  |  | X |  | 437 |
| **Froedtert Cancer Center Medical College of Wisconsin** | FoundationACT |  | X |  | X |  | 62 |
|  | Foundation One CDx | X |  |  | X |  | 324 |
|  | Foundation One Liquid CDx |  | X |  | X |  | 70 |
| **City of Hope** | [FoundationACT](http://ashion.com/services/#panel-1) |  | X |  | X |  | 62 |
|  | Foundation One CDx | X |  |  | X |  | 324 |
|  | Foundation One Liquid CDx |  | X |  | X |  | 70 |
|  | GemExtra/Ashion (TGen) | X |  | X | X | X | 19,396 |
|  | [Guardant360](https://www.therapyselect.de/sites/default/files/downloads/guardant360/guardant360_specification-sheet_en.pdf) CDx |  | X |  | X |  | 74 |
|  | Invitae Germline (unspecified) |  |  | X |  | X | N/A |
|  | Liquid Genomics (prostate) |  | X |  | X |  | N/A |
| **Tisch Cancer Institute Icahn School of Medicine at Mount Sinai** | Sema4 (Mount Sinai-affiliated) | X |  |  | X |  | 161 |
|  | [Sema4 Hereditary](https://cdn1.sema4.com/wp-content/uploads/Sema4-Signal-Hereditary-Cancer-Panel-Guide-1.pdf) Cancer Panel |  |  | X |  | X | 112 |
|  | [Foundation One Liquid](https://assets.ctfassets.net/w98cd481qyp0/7IpxsdNOTHT8SbzYLd6MV7/828dcc78b1fb4750680723819615fb03/F1L_TechnicalInformation_10__1_.pdf) CDx |  | X |  | X |  | 70 |
| **University of California San Francisco** | Ambry (Unspecified) |  |  | X |  | X | N/A |
|  | Color Hereditary Cancer Test |  |  | X |  | X | 30 |
|  | Datar Cancer Genetics Limited Cancertrack |  | X |  | X |  | N/A |
|  | FoundationACT |  | X |  | X |  | 62 |
|  | Foundation One CDx | X |  |  | X |  | 324 |
|  | Foundation One Liquid CDx |  | X |  | X |  | 70 |
|  | Invitae 85 Gene Panel |  |  | X |  | X | 85 |
|  | Invitae 87 Gene Panel |  |  | X |  | X | 87 |
|  | Invitae 90 Gene Panel |  |  | X |  | X | 90 |
|  | Invitae Common Hereditary Cancers Panel |  |  | X |  | X | 47 |
|  | Invitae Germline (unspecified) |  |  | X |  | X | N/A |
|  | Invitae Multi-Cancer Panel |  |  | X |  | X | 84 |
|  | Invitae Prostate Cancer Panel (19 gene panel) |  |  | X |  | X | 19 |
|  | Strata NGS | X |  |  | X |  | 437 |
|  | UCSF 500 Cancer Gene Panel | X |  |  | X |  | 529 |
| **Stephenson Cancer Center University of Oklahoma** | FoundationACT |  | X |  | X |  | 62 |
|  | Foundation One CDx | X |  |  | X |  | 324 |
|  | Foundation One Liquid CDx |  | X |  | X |  | 70 |
|  | Invitae Germline (unspecified) |  |  | X |  | X | N/A |
|  | Invitae Common Hereditary Cancers Panel |  |  | X |  | X | 47 |
|  | Myriad Germline (unspecified) |  |  | X |  | X | N/A |
|  | Myriad MyRisk Hereditary Cancer |  |  | X |  | X | 48 |
|  | PROfound HRR Assay (clinical trial assay) | X |  |  | X |  | 15 |
| **Karmanos Cancer Institute  Wayne State University** | Ambry CancerNext |  |  | X |  | X | 36 |
|  | Caris | X |  |  | X |  | 592 |
|  | Guardant360 |  | X |  | X |  | 74 |
|  | Invitae Germline (unspecified) |  |  | X |  | X | N/A |
|  | Invitae Hereditary Cancers Panel |  |  | X |  | X | 47 |
| **Henry Ford Cancer Institute** | Ambry CancerNext (34 gene panel) |  |  | X |  | X | 34 |
|  | Ambry CancerNext (36 gene panel) |  |  | X |  | X | 36 |
|  | Ambry CustomNext (genes selected by clinican) |  |  | X |  | X | Up to 91 |
|  | Ambry ProstateNext |  |  | X |  | X | 14 |
|  | Caris (unspecified) | X |  |  | X |  | 592 |
|  | Foundation One CDx (324 gene panel) | X |  |  | X |  | 324 |
|  | Foundation One CDx (395 gene panel) | X |  |  | X |  | 395 |
|  | Guardant360 CDx |  | X |  | X |  | 74 |
|  | Henry Ford BRCA1/BRCA2 |  |  | X |  | X | 2 |
|  | Henry Ford Comprehensive Solid Tumor Cancer Panel | X |  |  | X |  | 170 |
|  | Henry Ford Comprehensive Solid Tumor Fusion 50 Gene Panel | X |  |  | X |  | 50 |
|  | Henry Ford Hereditary Multi-Cancer Risk Assessment Panel |  |  | X |  | X | 39 |
|  | Invitae Hereditary Cancer Panel |  |  | X |  | X | 47 |
|  | Invitae Prostate Cancer Panel (12 gene panel) |  |  | X |  | X | 12 |
|  | Tempus xF (77 gene panel) |  | X |  | X |  | 77 |
|  | Tempus xF |  | X |  | X |  | 105 |
|  | Tempus xO (1711 gene panel) | X |  | X | X | X | 1711 |
|  | Tempus xO (1714 gene panel) | X |  | X | X | X | 1714 |
|  | Tempus xT (595 gene panel) | X |  |  | X |  | 595 |
|  | Tempus xT (596 gene panel) | X |  |  | X |  | 596 |
|  | Tempus xT (648 gene panel) | X |  |  | X |  | 648 |
|  | TruSeq Cancer Panel | X |  |  | X |  | 48 |
| **Tulane University** | Caris | X |  |  | X |  | 592 |
|  | Circulogene Theranostics Personalized Gene Profile |  | X |  | X |  | 50 |
|  | Color Hereditary Cancer Test |  |  | X |  | X | 30 |
|  | Foundation One CDx | X |  |  | X |  | 324 |
|  | Foundation One Liquid CDx |  | X |  | X |  | 70 |
|  | Guardant360 |  | X |  | X |  | 74 |
|  | Invitae Germline (unspecified) |  |  | X |  | X | N/A |
|  | Invitae Multi-Cancer Panel (81 genes) |  |  | X |  | X | 81 |
|  | Invitae Multi-Cancer Panel (83 genes) |  |  | X |  | X | 83 |
|  | Invitae Multi-Cancer Panel (84 genes) |  |  | X |  | X | 84 |
|  | Myriad MyRisk Hereditary Cancer |  |  | X |  | X | 48 |
|  | Resolution Bioscience ctDx (HRD) |  | X |  | X |  | N/A |
| **Holden Comprehensive Cancer Center University of Iowa** | Foundation One CDx | X |  |  | X |  | 324 |
|  | Guardant360 |  | X |  | X |  | 74 |
|  | Invitae Germline (unspecified) |  |  | X |  | X | N/A |
|  | Tempus xF |  | X |  | X |  | 105 |
